# Biocide-mediated corrosion of coiled tubing

**DOI:** 10.1371/journal.pone.0181934

**Published:** 2017-07-26

**Authors:** Mohita Sharma, Dongshan An, Tao Liu, Tijan Pinnock, Frank Cheng, Gerrit Voordouw

**Affiliations:** 1 Petroleum Microbiology Research Group, Department of Biological Sciences, University of Calgary, Alberta, Canada; 2 Department of Mechanical and Manufacturing Engineering, University of Calgary, Calgary, Alberta, Canada; The University of Akron, UNITED STATES

## Abstract

Coiled tubing corrosion was investigated for 16 field water samples (S5 to S20) from a Canadian shale gas field. Weight loss corrosion rates of carbon steel beads incubated with these field water samples averaged 0.2 mm/yr, but injection water sample S19 had 1.25±0.07 mm/yr. S19 had a most probable number of zero acid-producing bacteria and incubation of S19 with carbon steel beads or coupons did not lead to big changes in microbial community composition. In contrast other field water samples had most probable numbers of APB of 10^2^/mL to 10^7^/mL and incubation of these field water samples with carbon steel beads or coupons often gave large changes in microbial community composition. HPLC analysis indicated that all field water samples had elevated concentrations of bromide (average 1.6 mM), which may be derived from bronopol, which was used as a biocide. S19 had the highest bromide concentration (4.2 mM) and was the only water sample with a high concentration of active bronopol (13.8 mM, 2760 ppm). Corrosion rates increased linearly with bronopol concentration, as determined by weight loss of carbon steel beads, for experiments with S19, with filtered S19 and with bronopol dissolved in defined medium. This indicated that the high corrosion rate found for S19 was due to its high bronopol concentration. The corrosion rate of coiled tubing coupons also increased linearly with bronopol concentration as determined by electrochemical methods. Profilometry measurements also showed formation of multiple pits on the surface of coiled tubing coupon with an average pit depth of 60 μm after 1 week of incubation with 1 mM bronopol. At the recommended dosage of 100 ppm the corrosiveness of bronopol towards carbon steel beads was modest (0.011 mm/yr). Higher concentrations, resulting if biocide is added repeatedly as commonly done in shale gas operations, are more corrosive and should be avoided. Overdosing may be avoided by assaying the presence of residual biocide by HPLC, rather than by assaying the presence of residual surviving bacteria.

## Introduction

Flexible coiled tubing, which can be spooled onto a reel, is used in shale oil and shale gas operations for milling plugs from horizontal wells after hydraulic fracturing and other applications [1]. Recirculation of fluids in such closed loop operations has been associated with increased coiled tubing failures [2]. The presence of sand, fracturing chemicals, microbes, high rates of fluid injection and coiling-related material stresses may all contribute to high generalized and localized corrosion of coiled tubing [2–5], which is exposed to injection fluids internally and production fluids externally. When used in acidic and sour (H_2_S-containing) environments, coiled tubing is also susceptible to pitting and crevice corrosion, which may be a major cause of failure [3,6]. Microbially influenced corrosion (MIC), due to the action of sulfate reducing bacteria, has been observed in coiled tubing biased weld points [4], necessitating the use of biocides [7].

We have described an integrated approach of field water sample analyses, which included determination of water chemistry, most probable numbers of microorganisms, microbial community composition and general corrosion rates to determine possible causes for high corrosion rates in coiled tubing [8]. Coiled tubing fluid samples were collected from a milling operation where plugs were being milled out to prepare the well for production in a shale gas field near Grande Prairie, Alberta, Canada. The coiled tubing operators were facing issues of recurrent corrosion failures and hence the reasons for these failures were being investigated. From our earlier investigations, we concluded that acid-producing bacteria, including *Pseudomonas*, may have contributed to corrosion by fermentation of guar gum (a frequently used proppant in hydraulic fracturing) to organic acids, which lowers the pH of coiled tubing fluids. Direct addition of acetic acid to coiled tubing fluid samples, lowering the pH to 3.5, did indeed increase general corrosion rates from 0.12–0.20 mm/yr to 0.18–1.04 mm/yr [8]. However, this could not explain the already high general corrosion rate of 1.31 mm/yr observed for injection water sample S19, which had a pH of 6.1. This field water sample had zero counts of acid producing bacteria and sulfate reducing bacteria [8], prompting a reexamination of potential causes of this high corrosion rate as described in the current paper.

## Materials and methods

### Sample and field information

**Fig 1:**
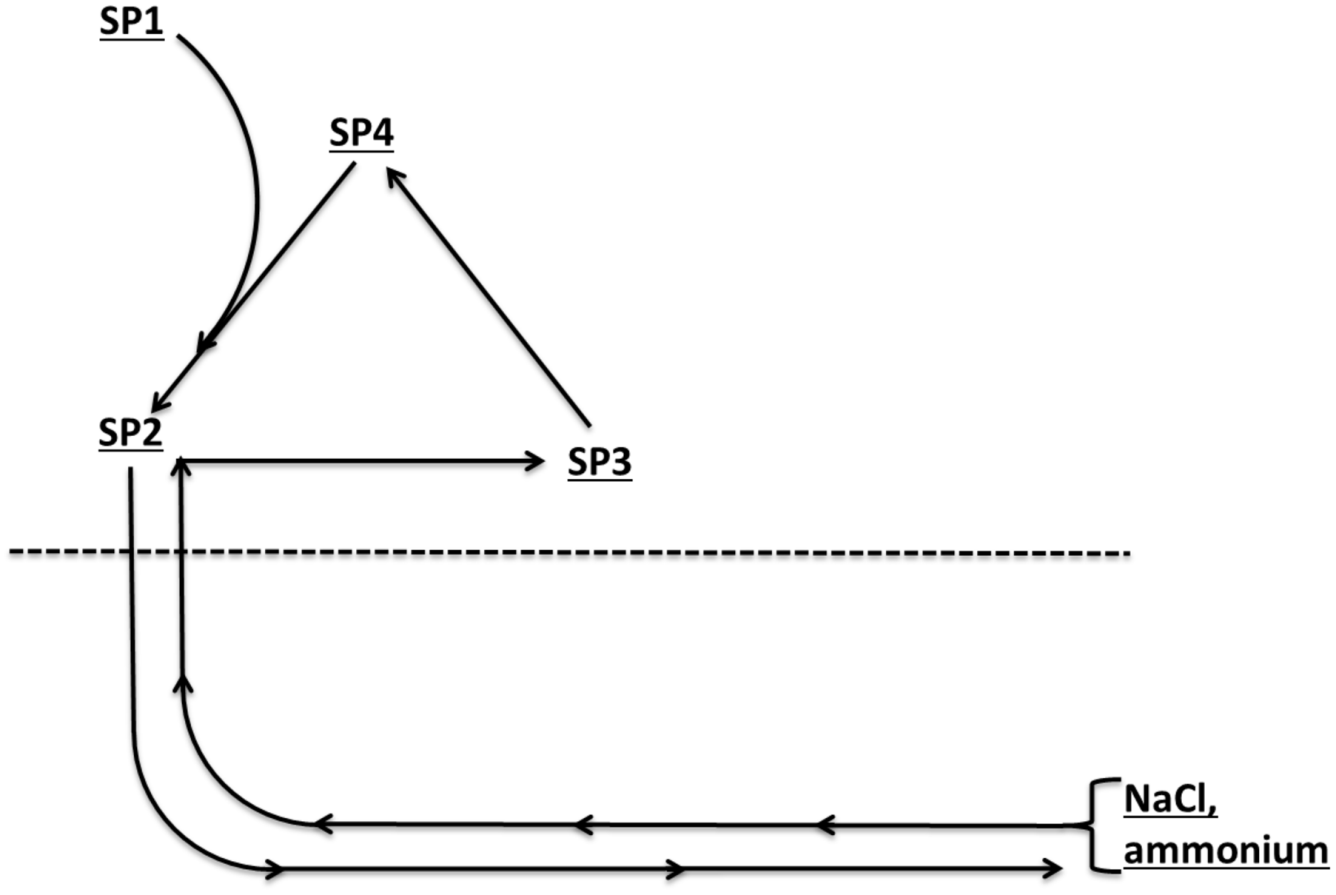
Schematic of field water sample types obtained from a CT operation. SP1, source water; SP2, injection water; SP3, produced water prior to passing through test separators; SP4, produced water after passing through test separators. The dotted line represents the surface. Injection water (rightward pointing arrows) in the CT will pick up NaCl and ammonium when water flows past the milling bit and returns to surface as produced water (leftward pointing arrows) in the CT annulus (the space between the production tubing and the CT).

Sixteen field water samples (S5 to S20) from a coiled tubing milling operation in a shale gas field near Grande Prairie, Alberta, Canada were collected in autoclaved sterile 1 L plastic bottles. These were either SP1, SP2, SP3 or SP4 type, as explained in Fig 1, and were collected over 3 days of operation (Table 1), in which coiled tubing was used to mill out the plugs present in a horizontal well successively to clear the well for gas production after fracturing. Fig 1 indicates the recirculation pattern of water during this closed loop operation, in which water was reused to mill through multiple plugs. Source water from the fresh water tank (SP1) was mixed with separated produced water (SP4) to give injection water (SP2), which returned to the surface as produced water or flow back water (SP3), which was passed through a separator to separate sand, water and gas condensate. Separated water (SP4) was then again mixed with source water to start a new 12 h cycle. SP1, SP2 and SP4 were stored in tanks, which were subject to addition of 0.1 kg/m^3^ (100 ppm) of the biocide bronopol at the start of every cycle, i.e. a cumulative dose of 600 ppm (3 mM) could result in 6 cycles over the 3-day period. Due to perceived microbial contamination in the source water tank on day 2, additional amounts (unspecified) of bronopol were added. The field water samples were received within 24 h of collection and transferred to an anaerobic hood with an atmosphere of 90% N_2_, 10% CO_2_ (N_2_-CO_2_).

**Table 1:** Chemical and microbial properties, as well as corrosion rates of carbon steel beads for 16 water samples S5 to S20 from a coiled tubing milling operation in a shale gas field in Alberta, Canada. ^1)^ The table is ranked by day of operation and sample type.

Day^2)^	Sample ID	Sample type	pH	NaCl (Meq)	Ammonium (mM)	Bromide (mM)	Corrosion rate (mm/yr)	log APB/mL^3)^
1	S5, S6	SP1	6.58	0.00	0.02	0.09	0.22	1.69
3	S18	SP1	6.66	0.00	0.10	0.01	0.31	2.38
2	S10, S13	SP2	6.38	0.15	2.82	0.83	0.11	3.27
3	S15, S16	SP2	6.47	0.39	5.79	2.02	0.21	7.18
3	S19	SP2	6.14	0.43	0.04	4.20	1.31	0.00
1	S7, S8	SP3	6.32	0.00	0.20	0.09	0.21	3.19
2	S9, S12	SP3	6.64	0.22	3.38	1.06	0.14	2.99
3	S20	SP3	6.39	0.46	7.67	2.90	0.17	6.38
2	S11	SP4	6.53	0.20	3.33	2.50	0.21	6.97
3	S14, S17	SP4	6.28	0.40	6.21	1.82	0.11	5.38

^1)^Data for pH, NaCl (Meq), ammonium (mM) and corrosion rate (mm/yr) are from [8]; data for bromide (mM) are from this study; averages are given when two field water samples are listed.

^2)^Day of operation on which field water sample was taken.

^3)^ Logarithm of the most probable number (MPN/mL) of acid-producing bacteria are from [8].

#### Water chemistry tests and most probable number of acid-producing bacteria

Aliquots of 20 mL from vigorously mixed field water samples were used for water chemistry tests. The pH, salinity expressed as molar equivalent (Meq) of NaCl, and the concentration of ammonium were determined previously as well as the most probable number of acid-producing bacteria [8]. The concentration of bromide was determined using a Waters high performance liquid chromatograph with a conductivity detector (Waters 423) and an IC-PAK anion column (4 x 150 mm, Waters), eluted with borate/gluconate buffer at a flow rate of 2 mL/min.

### Weight loss corrosion tests

The use of ASTM a36 carbon steel ball bearings (referred to as beads) for measuring general corrosion rates has been described recently [9]. These have a very homogeneous size (55.0±0.3 mg;Ø = 0.238 cm). Coupons (1.3x0.8x0.4 cm), cut from coiled tubing steel, were used both for incubations and for electrochemical studies, described below. NACE RP0775-2005 protocol was followed for the pretreatment of carbon steel beads and coupons. This included exposure for 2 min to dibutylthiourea-HCl solution, sodium bicarbonate solution, deionized water and a final wash with acetone. Beads or coupons were air-dried and weighed using an analytical balance before the initiation of the experiments [8].

For corrosion testing, 50 mL of each field water sample, collected from coiled tubing milling operation site, were placed into 120 mL serum bottles containing 5 pre-weighed beads or 3 coiled tubing coupons in a N_2_-CO_2_ atmosphere. These were then sealed with rubber stoppers and aluminum crimps and incubated at 30°C on a rotary shaker at 150 rpm. Incubations were for 32 days unless stated otherwise. At the end of incubation, the beads and the coupons were again treated with the NACE RP0775-2005 protocol, air-dried and reweighed to measure the weight loss and calculate corrosion rate (R_*corr*_) in mm/yr:

$${\text{R}}_{corr}=87600\;\times \;\left(\frac{\Delta W}{D\;\times A\times T}\right)$$ (1)

Where, Δ*W* is the weight loss (g) at the end of incubation, D is the density of carbon steel (7.85 g/cm^3^), A is the surface area of the beads (5×0.1781 = 0.8905 cm^2^) or coupons (3x3.76 = 11.28 cm^2^) and T is the incubation time (h).

### Electrochemical corrosion tests

Coiled tubing carbon steel coupons (1.3x0.8x0.4 cm) were ground with silicon carbide papers (from 120 to 1000 grade) using an automated polisher (Buchler Ltd) and then degreased with acetone, washed with deionized water and dried. Coupons were then embedded in an epoxy resin leaving an exposed surface area of 1 cm^2^ and leaving one end connected to a processed solid wire (wire-300, VHU 18 GA, NTE Electronics, Inc.). The working electrode (WE) was kept in a coupon holder, connected to a 2 L tank containing the test concentration of bronopol (0, 1, 1.5, 2 or 2.5 mM) in Coleville Synthetic Brine K (CSBK) medium, and this solution was recirculated for 24 hours in an N_2_-CO_2_ atmosphere. The electrochemical tests were subsequently performed on these coupons using a three-electrode cell set-up, with the coiled tubing coupon used as the WE, a graphite rod as the counter electrode (CE), and Ag/AgCl/3.5 M KCl as the reference electrode (RE) in an N_2_-CO_2_ atmosphere. Non-destructive methods like electrochemical impedance spectroscopy (EIS) provide mechanistic information on the corrosion process through the use of small signals, which do not disturb the electrode properties to be measured [10,11]. EIS was performed with Gamry Instrument 600 under open circuit potential (OCP) conditions from 10^−2^ to 10^5^ Hz with a sinewave amplitude of 10 mV. The experimental EIS spectra were interpreted on the basis of equivalent electrical analogs using the ZSimpWin software to obtain fitting parameters. To obtain more precise fitting results, the capacitance elements (C) in the equivalent electrical circuits (EECs) employed were replaced with constant phase elements (CPE) due to non-ideal behavior in the form of a depressed semi-circle [12]. The impedance of CPE is defined by:

$$Z\left(j\omega \right)={\left(Y_{0}\right)}^{-1}{\left(j\omega \right)}^{-n}$$ (2)

where *Y*_*0*_ is the parameter related to capacitance, *j* is the imaginary unit, *n* is the constant phase angle of CPE (0≤n≤1, rad) and *ω* is the angular frequency (*ω = 2πf*, *f* is the frequency, rad/s). For *n* = 1, the CPE is pure capacitance. For a capacitance element, a decrease in the value of n from n = 1 indicates metal surface heterogeneity [12–15].

Following EIS, potentiodynamic polarization curves were measured on the coiled tubing electrode. The potential scan was performed from -200 to +200 mV with respect to the OCP at a sweep rate of 5 mV/sec to derive the cathodic and anodic Tafel slopes *b*_*c*_ and *b*_*a*_. The corrosion current density i_corr_ was calculated using the Stern-Geary equation:

$$i_{corr}=\frac{b_{c}\times \;b_{a}}{2.303R_{p}\;\left(b_{c}-b_{a}\right)}$$ (3)

where *R*_*p*_ represents the polarization resistance calculated from the linear region of the polarization curves [16].

### Profilometry measurements

Polished coiled tubing coupons embedded in an epoxy resin with an exposed surface area of 1 cm^2^ were incubated with 100 mL CSBK medium with different concentrations of bronopol (0–2.5 mM) for one week in an N_2_-CO_2_ atmosphere in sterile Nalgene bottles. These were kept on a rotary shaker at 150 rpm and incubated at 30°C. At the end of one week, the coupons were cleaned using NACE RP0775-2005 protocol described earlier and dried under N_2_ gas stream. The surface profile of the coupons was subsequently determined using optical profilometer (Zygo Zescope).

### Analysis of bronopol concentration

The quantitation of bronopol (2-bromo-2-nitropropane-1,3-diol) was performed using a reversed-phase HPLC separation method with UV spectrophotometric detection at 210 nm [17]. An Alltima C18 column of 250 x 4.6 mm (Grace Davison) was used. Bronopol (Sigma) was used for the preparation of standard stock solutions. A mixture of methanol (5%), water (95%) and 1 M orthophosphoric acid (0.1% v/v) was used as the mobile phase at a flow rate of 0.5 mL/min.

### Microbial community analysis

Genomic DNA was isolated from incubations of field water samples with carbon steel beads or coiled tubing coupons performed for analysis of general corrosion rates by weight loss. This included incubations in which field water sample S19 was used as is, or was used following filtration through an 0.2 μm Millipore filter (S19_F). Incubations in which these samples were diluted with CSBK medium or were amended with additional bronopol were also used. For all incubations 50 mL was centrifuged (15 min, 12000 rpm) and the pellet used for DNA isolation. For incubations with coupons, one coupon was removed and scraped with the scrapings being collected in a microcentrifuge tube. Following centrifugation, the pellets were used for DNA isolation. DNA isolation was performed using the FAST DNA^®^ Spin kit for soil as per the manufacturer’s instructions (MP Biomedicals). The concentration of the isolated DNA was quantified using the Qubit fluorometer (Invitrogen) with the Quant-iT^™^ dsDNA HS assay kit (Invitrogen). The DNA templates were cleaned using the DNA Clean and Concentrator^™^ kit (Zymo Reseach). PCR amplification was done with a two-step process with triplicate reactions of 20 μl each in each step. The amplified products were pooled and cleaned using the QIAquick PCR purification kit (Qiagen). The quality of the amplified products was analyzed on a 1.5% (w/v) agarose gel and the DNA concentration was quantified fluorimetrically. Primer dimers were removed with the Select a Size DNA Clean and Concentrator^™^ kit (Zymo Research). In the first step, non-barcoded primers (926Fi5: 5’-TCGTCGGCAGCGTCAGATGTGTATAAGAGACAGAAACTYAAAKGAATWGRCGG-3’ and 1392Ri7:5’GTCTCGTGGGCTCGGAGATGTGTATAAGAGACAGACGGGCGGTGWGTRC-3’) were used for 25 cycles, followed by a second PCR with barcoded primers (P5-S50X-OHAF and P7-N7XX-OHAF) for 8 cycles. The barcoded forward primer (P5-S50X-OHAF) contained a 29-nucleotide 5’ Illumina sequencing adaptor (P5, AATGATACGGCGACCACCGAGATCTACAC), an 8 nucleotide index S50X and a 14-nucleotide forward overhang adaptor (OHAF, TCGTCGGCAGCGTC). The barcoded reverse primer (P7-N7XX-OHAF) had a 24 nucleotide 3’ Illumina sequencing adaptor (P7, CAAGCAGAAGACGGCATACGAGAT), an eight nucleotide index N7XX and a 14 nucleotide reverse overhang adaptor (OHAF, GTCTCGTGGGCTCGG). In case of samples, which had DNA concentrations less than 0.1 ng/μl, a boost PCR was performed. The cycling conditions for the 1st PCR were 95°C for 5 min, followed by 25 cycles of 95°C for 30 s, 55°C for 30 s, 72°C for 60 s, 72°C for 10 min. The cycling conditions for the 2nd PCR reaction were 95°C for 3 min, followed by 8 cycles of 95°C for 30 s, 55°C for 30 s, 72°C for 90 s and 72°C for 10 min. Purified and concentrated PCR products were diluted to 2 ng/μl and were sequenced using the 300PE (paired-end) MiSeq protocol on an Illumina Miseq system at the Energy Bioengineering Group, Department of Geosciences, University of Calgary. The 300PE reads were merged into single reads using PEAR 0.9.6 and further processed using MetaAmp, a 16S rRNA data analysis pipeline, developed by the Energy Bioengineering Group (http://ebg.ucalgary.ca/metaamp)[18]. Raw 16S rRNA amplicon sequences are available from the Sequence Read Archive (SRA) at NCBI (Accession number: SAMN05467017, http://www.ncbi.nlm.nih.gov/sra/SAMN05467017). The sequencing data retrieved were further clustered into operational taxonomic units (OTUs) at a taxonomic distance of 3%. The distance between the communities was calculated using the Bray Curtis coefficient in the Mothur software and UPGMA (unweighted pair group method algorithm) was used for clustering the samples into a dendrogram, which was subsequently visualized using MEGA 5.2.2 software [19].

## Results and discussion

### Water chemistry and most probable number of microorganisms (MPNs)

**Fig 2:**
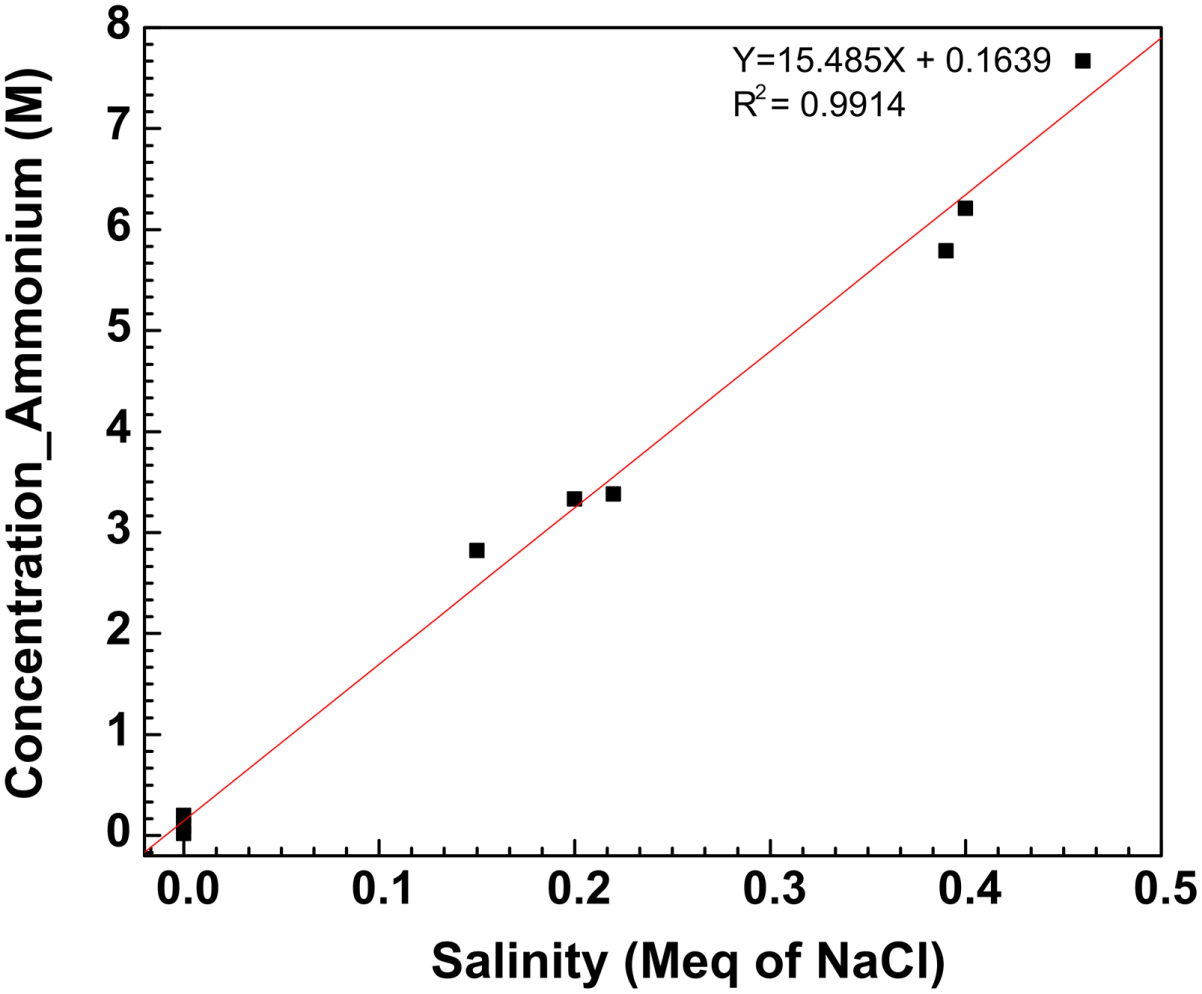
Relation between salinity (Meq of NaCl) and ammonium concentration (mM) for the field water samples. Some data are averages for two samples as indicated in Table 1.

The water chemistry data obtained previously [8] for field water samples S5 to S20 were used to generate Table 1. Data for the field water samples of the same type (SP1, SP2, SP3 or SP4), collected on day 1, 2 or 3, were averaged e.g. for S5 and S6, which are both SP1-type and were both collected on day 1 (Table 1). These data indicate that whereas pH was constant, the salinity and ammonium concentrations increased with the number of times the water was reused (2 cycles/day). The average salinities for all SP2-, SP3- and SP4-type samples on days 1, 2 and 3 were 0, 0.18 and 0.41 Meq of NaCl, respectively, whereas the average ammonium concentrations were 0.2, 3.2 and 5.3 mM, respectively. Salinity and ammonium concentration had a strong linear correlation (Fig 2; r^2^ = 0.99; p <0.001). Their increase with time (number of cycles of reuse) was due to increased exposure to subsurface fracturing water, which had high salinity and ammonium (Fig 1).

The field water samples had modest concentrations of sulfate (up to 3 mM) and iron (up to 1 mM), as well as low concentrations of sulfide, acetate and propionate of up to 0.1, 0.6 and 0.5 mM, respectively [8]. The field water samples had up to 11 g/L of sand and suspended solids and up to 10^7^ acid-producing bacteria/mL, able to actively ferment guar gum to organic acids [8]. No acid-producing bacteria were detected in injection water sample S19 (Table 1, [8]). Sulfate-reducing bacteria able to actively reduce sulfate with lactate as electron donor, were not detected in any of the field water samples [8].

### High corrosion rate of sample S19

General weight loss corrosion rates, determined by incubation of 50 mL of field water samples S5 to S20 with 5 carbon steel beads, were 0.1 to 0.3 mm/yr for all samples except S19, which had 1.31 mm/yr (Table 1). As indicated previously [8], weight loss corrosion rates determined for coiled tubing coupons were lower (0.02 to 0.19 mm/yr) with S19 having again the highest weight loss corrosion rate (0.19±0.003 mm/yr). The seven-fold difference in values between beads and coupons could be due to different grades of steel, to a different surface to volume ratio of beads (12.6 cm^-1^) versus coupons (9.0 cm^-1^) or to the different weights of steel added to the assay medium, which was 0.275 g for beads versus 9.80 g for coupons. Weight loss corrosion rates of carbon steel beads decreased strongly with an increasing number of beads per unit volume [9]. Weight loss corrosion rates reported in the remainder of this section are all for carbon steel beads.

S19 had most probable numbers of zero for acid-producing bacteria and sulfate-reducing bacteria (Table 1, [8]), which makes microbially influenced corrosion unlikely as a possible reason for the high corrosion rate. To prove that microorganisms were not responsible for the high corrosion in S19, this injection water S19 sample was filtered through a 0.2 μm filter (Millipore) to remove microorganisms and this was also used for incubations (Table 2). Use of the filtrate in corrosion experiments, gave a corrosion rate of 1.28 mm/yr, whereas a corrosion rate of 1.17 mm/yr was found prior to filtration (Table 2, entries 1 and 9). Together with the value of 1.31 mm/yr obtained before (Table 1), the average corrosion rate for S19 or its filtrate was 1.25±0.07 mm/yr (N = 3). Dilution of S19 or its filtrate with Coleville Synthetic Brine K (CSBK) medium decreased the corrosion rate proportionally (Table 2, entries 1 to 4 and 9 to 12). S19 had 4.2 mM of bromide, a possible degradation product of bronopol, the highest concentration of any of the field water samples (Tables 1 and 2, entry 1). Determination of the concentration of active bronopol by reversed-phase HPLC indicated the presence of 13.8 mM (2760 ppm) in S19 (Table 2, entry 1). No active bronopol could be detected in any of the other field water samples, although most of these also had an elevated concentration of bromide (Table 1: S11, 2.5 mM; S15 and S16, 2.0 mM; S20, 2.9 mM). The average bromide concentrations in field water samples on days 1, 2 and 3, calculated from the data in Table 1, were 0.1, 1.3 and 2.1 mM, respectively. The increase may be due to application of 100 ppm bronopol (0.5 mM) at each cycle of water reuse. Assuming conversion of bronopol to bromide and two cycles of water reuse per day and no loss of bromide during water injection and production cycles, bromide concentrations of 0.5, 1.5 and 2.5 mM are expected on days 1, 2 and 3.

**Table 2:** General corrosion rates of carbon steel beads incubated for 30 days with S19, with S19 passed through an 0.2 μm filter (S19_F) or with Coleville Synthetic Brine K (CSBK) medium. Dilutions of S19 and of S19_F with CSBK were also used. In addition to the bronopol present in S19 (13.8 mM), bronopol was also added as indicated.

#	S19 (mL)	S19_F (mL)	CSBK (mL)	Bronopol added (mM)	Bromidebefore (mM)^1)^	Active bronopol (mM)^2)^	Corrosion rate (mm/yr)^3)^	Bromideafter (mM)^1)^	Bromide after (mM) theoretical^4)^
1	30		0	0	4.2	13.8	1.17±0.032	18.47	18
2	22.5		7.5	0	3.15	10.35	0.86±0.046	13.83	13.5
3	15		15	0	2.1	6.9	0.64±010	9.34	9
4	7.5		22.5	0	0.85	3.45	0.34±0.028	4.34	4.3
5	30		0	10	4.2	23.8	1.62±0.011	29.35	28
6	22.5		7.5	7.5	3.15	17.85	1.2±0.009	22.05	21
7	15		15	5	2.1	11.9	0.92±0.022	14.51	14
8	7.5		22.5	2.5	0.85	5.95	0.36±0.021	5.63	6.8
9		30	0	0	4.2	13.8	1.28±0.005	21.9	18
10		22.5	7.5	0	3.15	10.35	1.07±0.029	16.39	13.5
11		15	15	0	2.1	6.9	0.69±0.009	10.78	9
12		7.5	22.5	0	0.85	3.45	0.52±0.008	7.14	4.3
13^5)^		±cells	30	10	0	10	0.71±0.05	ND	10
14			30	2.5	0	2.5	0.2±0. 0.03	2.58	2.5
15			30	2	0	2	0.18±0.021	1.86	2
16			30	1.5	0	1.5	0.15±0.005	1.34	1.5
17			30	1	0	1	0.09±0.007	0.90	1
18			30	0.5	0	0.5	0.07±0.028	0.48	0.5
19^5)^		±cells	30	0	0	0	0.02±0.007	0	0

^1)^Bromide concentration before and after the corrosion experiment, as determined by anion HPLC.

^2)^Active bronopol concentration. The value in S19 (13.8 mM, 2760 ppm) was determined by reverse phase HPLC. All other values were calculated.

^3)^Average ± SD for duplicate incubations with 5 carbon steel beads each.

^4)^Sum of the concentrations of active bronopol and of bromide before incubation, assuming release of 1 bromide per active bronopol during the incubation.

^5)^These incubations were done both in the absence and presence of cells and solids obtained by filtration of 30 mL S19 through an 0.2 μm filter; “+cells” means that the filter was added to these incubations.

**Fig 3:**
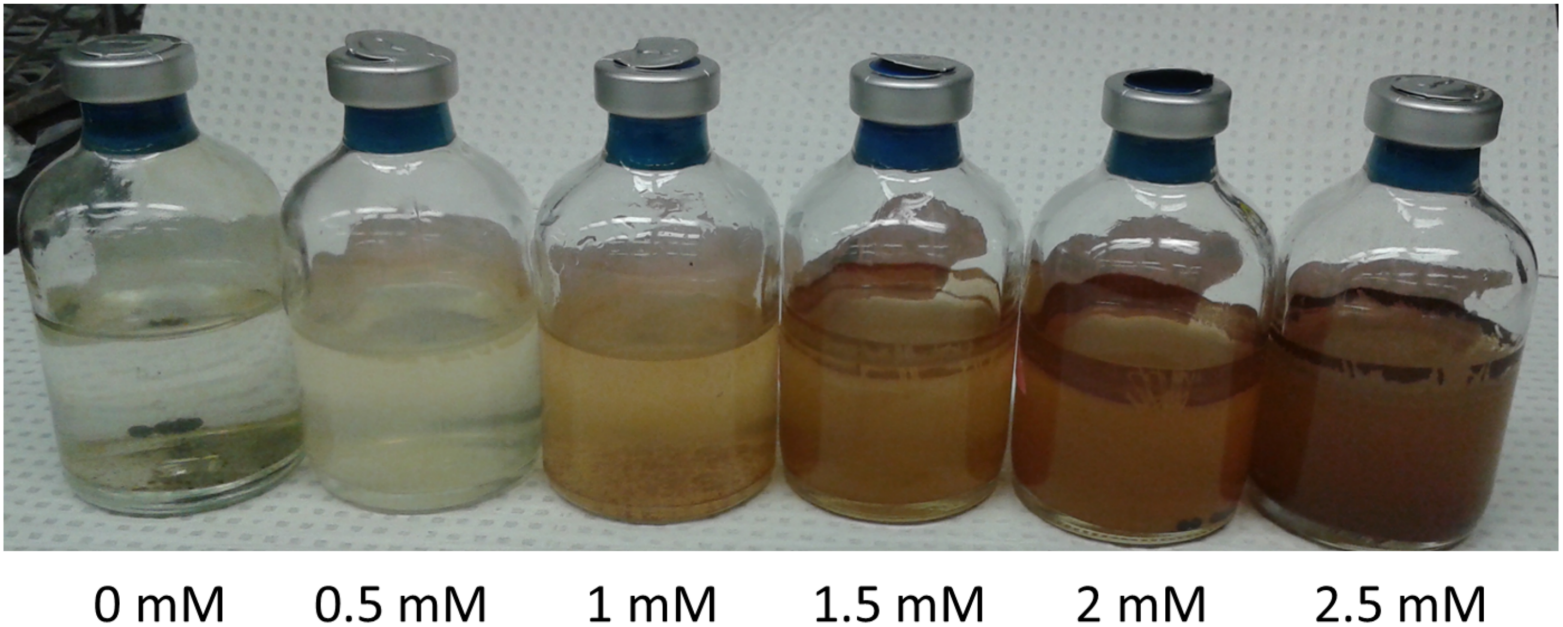
Incubation of carbon steel beads in serum bottles (five per bottle) with an N_2_-CO_2_ headspace and 30 mL of CSBK medium with 0 to 2.5 mM bronopol, as indicated. The image was taken after 30 days of incubation. All bottles were clear at the start of incubation.

In order to determine the corrosivity of bronopol, carbon steel beads were incubated with S19 amended with up to 10 mM (2000 ppm) of additional bronopol (Table 2: entries 5 to 8). Beads were also incubated with CSBK medium, to which up to 10 mM (2000 ppm) bronopol was added (Table 2: entries 13 to 19). Images for incubations 14 to 19 are shown in Fig 3. The active bronopol concentration and the bromide concentration at the start of these incubations are indicated in Table 2, together with the experimentally determined bromide concentrations at the end of the incubations. These are compared with the theoretical values, assuming production of one bromide per bronopol following its reaction with carbon steel or other mechanism of degradation during one month of incubation. Agreement between measured and predicted bromide concentrations was good (Table 2; r^2^ = 0.98; p <0.001). We used simple linear regression to test the relationship between the concentration of active bronopol (mM) and the corrosion rate (mm/yr). The F-statistic was calculated from the data and was compared to the theoretical F-distribution to calculate the p-value. This relationship was positive and statistically significant with F = 236.3, p < 0.001, r^2^ = 0.937).

**Fig 4:**
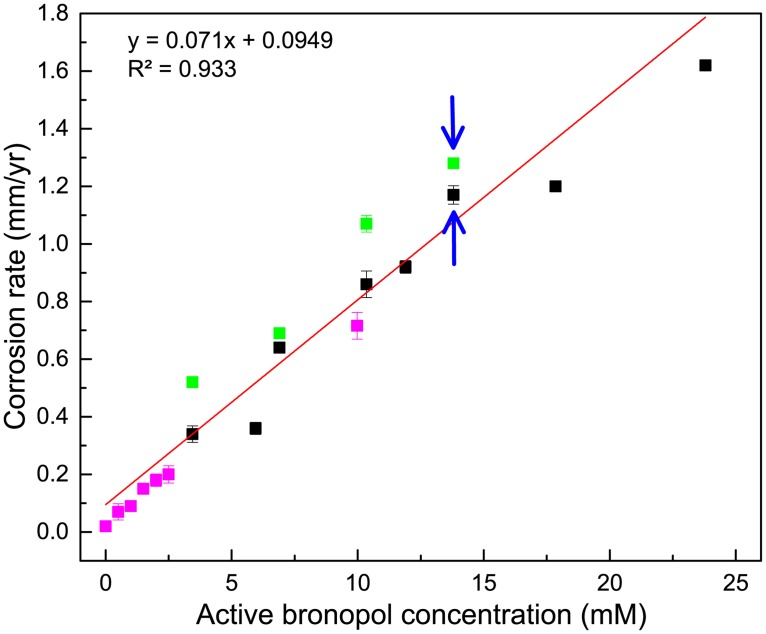
Corrosion rates of carbon steel beads as a function of active bronopol concentration. Beads (five per incubation) were incubated for 30 days with field injection water sample S19 (↑), with S19 with additional bronopol or with dilutions of S19 with CSBK (black symbols), with S19_F an 0.2 μm filtrate of S19 (↓) or with dilutions of S19_F with CSBK (green symbols), or with CSBK medium with 0 to 10 mM (0 to 2000 ppm) of bronopol added (pink symbols). Detailed conditions for all 19 incubations are given in Table 2. Data are averages for two incubations with 5 beads each; SD is shown when this exceeded the size of the symbols.

Plotting the measured corrosion rate and the concentration of active bronopol indicated a linear relationship for all 19 incubations listed in Table 2 (Fig 4). The different experimental conditions have been color-coded differently (Table 2: 1–8 black, 9–12 green and 13–19 pink). Altogether these data indicate that the high corrosion rate in S19 was caused by the presence of an unusually high bronopol concentration of 18 mM (3600 ppm), the sum of the measured active bronopol (13.8 mM) and bromide (4.2 mM) concentrations.

An important question is why this excessively high dose was added. Because coiled tubing operations are complete within a short period of time (3 days), the possible presence of bacteria cannot be measured by classical most probable number assays, which typically require several weeks before microbial growth can be evaluated. The presence of potentially corrosive microbes is therefore measured by rapid assays, including the measurement of adenosine triphosphate (ATP). However, this mainstream metabolite is preserved by killing of cells with acid and its presence does, therefore, not need to indicate the presence of live cells. Falsely positive readings may lead to unneeded dosage increases. A strategy to avoid this would be to determine the biocide concentration directly. We have done this by an HPLC assay, which is rapid and can be adapted for use in the field. Addition is not needed if 100 ppm of active biocide is still detected.

### Electrochemical tests

**Fig 5:**
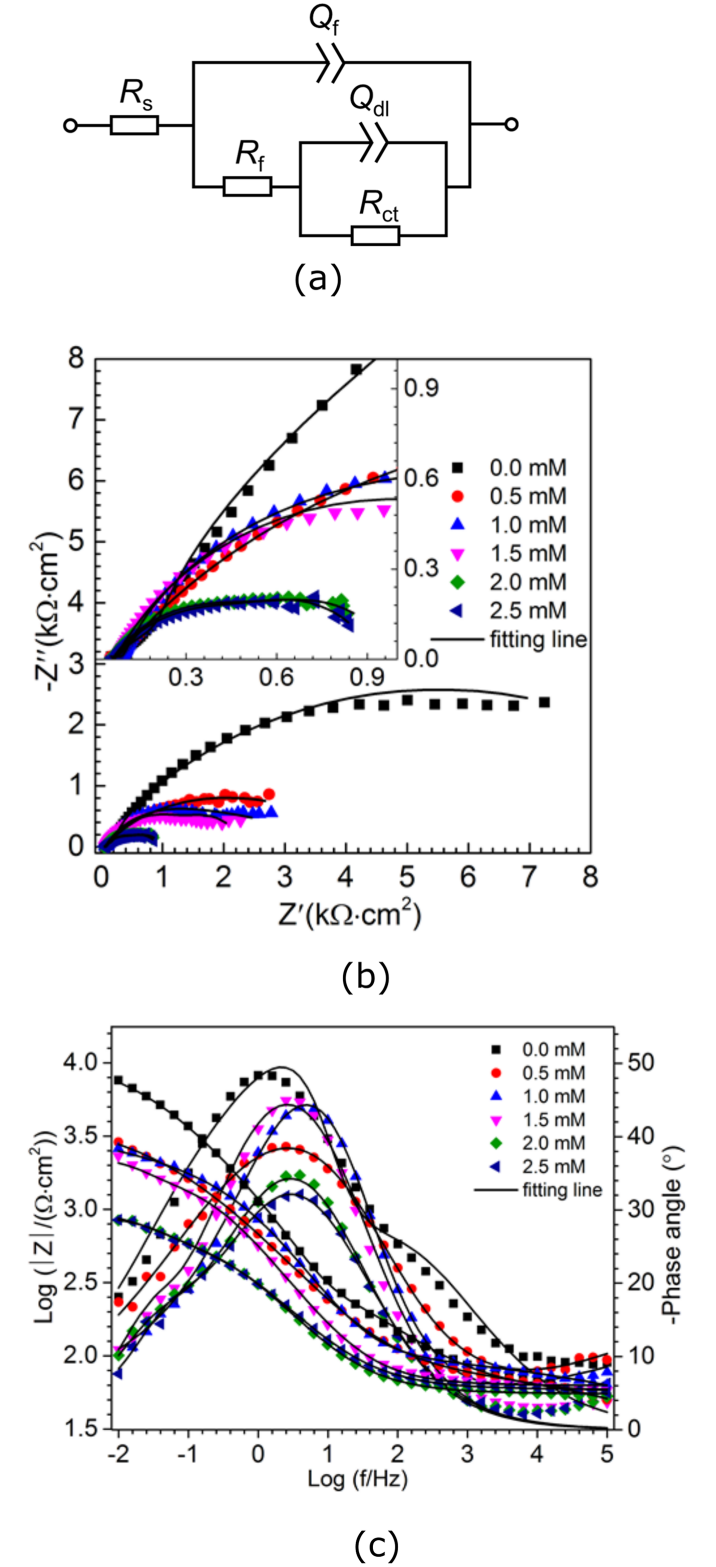
(a) Equivalent circuit used for simulating experimental impedance results (b) Nyquist and (c) Bode plots for EIS analysis of coiled tubing coupon WEs, incubated with 0 to 2.5 mM of bronopol in CSBK medium for 24 h. The reference electrode used was Ag/AgCl/3.5 M KCl.

Corrosion in the presence of bronopol was also evaluated for coiled tubing coupons as the working electrode (WE). The equivalent circuit (a) and the results of EIS analysis in the form of Nyquist (b) and Bode plots (c) of the coiled tubing coupon WE, following reaction with 0.5 to 2.5 mM bronopol for 24 h, are shown in Fig 5. Impedance spectra were analyzed using the equivalent circuit of Fig 5a, which consisted of solution resistance (*R*_*s*_), resistance and capacitance of the corrosion product film (*R*_*f*_ and *Q*_*f*_), and charge transfer resistance and double layer capacitance (R_ct_ and Q_dl_), respectively. EIS spectra were fitted using ZSimpWin software and the fitted electrochemical parameters are summarized in Table 3.

**Table 3:** Fitted EIS electrochemical parameters for CT coupon WEs incubated at different bronopol concentrations for 24 h. The tabulated data were derived from single incubations.

Bronopol (mM)	R_ct_ (kΩ·cm^2^)	Y_1_ ×10^−4^ (Ω^-1^·cm^2^ ·S^n^)	n_1_	R_f_ (kΩ·cm^2^)	Y_2_×10^−4^ (Ω^-1^·cm^-2^·S^n^)	n_2_	R_s_ (Ω·cm^2^)
0	10.8	2.967	0.956	0.4701	25.68	0.5295	55.77
0.5	4.782	3.475	0.5991	0.0857	2.36	0.2675	58.1
1.0	4.328	1.5557	0.7663	0.1064	3.208	0.2277	59.9
1.5	1.738	12.78	0.7978	0.5054	4.526	0.683	62.82
2.0	0.297	13.55	0.812	0.6452	8.489	0.6384	55.32
2.5	0.232	12.19	0.8999	0.6461	8.783	0.6125	59.13

The diameter of the Nyquist plots decreased (Fig 5b), as the bronopol concentration in the electrochemical cells was increased corresponding to a higher corrosion rate. Two time constants were observed in the Bode plots (Fig 5c), which indicate the formation of a corrosion product film on the surface of the coupon [20, 21]. Greater influence of charge transfer resistance (*R*_*ct*_) as compared to solution resistance (*R*_*s*_) was also observed as *R*_*ct*_ decreased from 10.8 to 0.232 kΩ·cm^2^ with increasing bronopol concentration from 0 to 2.5 mM (Table 3). There is a possibility of formation of porous corrosive products due to bronopol attack on the surface of CT coupons, which can further auto-catalyze pitting on the surface of the coupon and hence accelerate corrosion [22].

**Fig 6:**
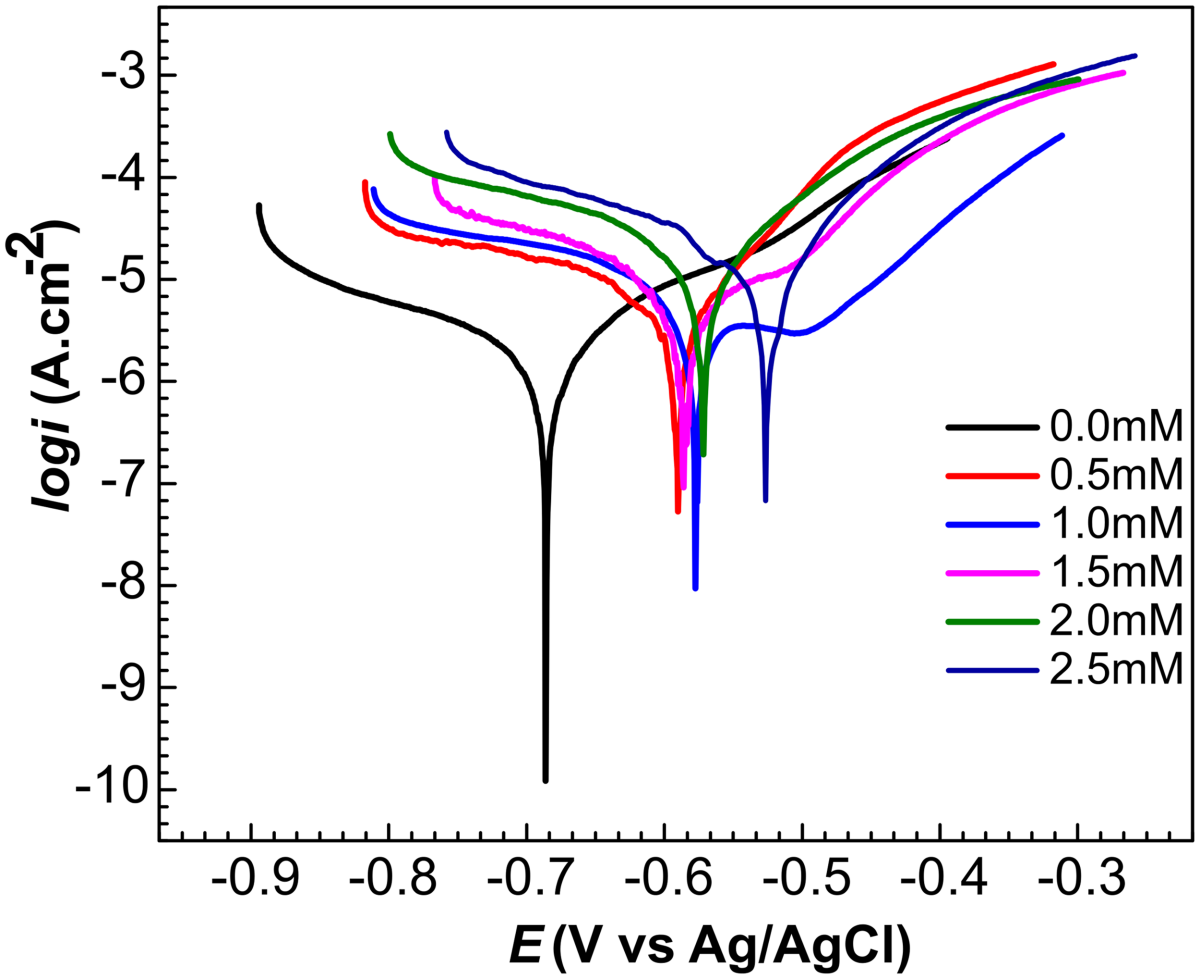
Potentiodynamic polarization curves for single incubations of coiled tubing coupon WEs following incubation with 0 to 2.5 mM of bronopol in CSBK for 24 h, as indicated. The reference electrode used was Ag/AgCl/3.5 M KCl.

Potentiodynamic polarization scanning indicated an increase in corrosion potential (E_corr_) from -0.68 to -0.52 V with respect to the Ag/AgCl reference electrode and an increase in the corrosion current (i_corr_) from 1.43 to 23 μA/cm^2^, as the bronopol concentration was increased from 0 to 2.5 mM (Fig 6, Table 4). This also demonstrates that the dose-dependent corrosiveness of bronopol, may be attributed to increased adsorption to the metal surface with increased concentration. The current density changed linearly with potential within ±10 mV of E_corr_ in the E-i curve. Results of potentiodynamic polarization scanning tests have been summarized in Table 4. The derived corrosion rates for coiled tubing coupons (Table 4: 0.016 to 0.264 mm/yr for bronopol concentrations from 0 to 2.5 mM) were similar to those obtained for carbon steel beads by weight loss (Table 2, entries 13 to 18: 0.02 to 0.20 mm/yr). However, this agreement is fortuitous, because actual weight loss corrosion rates for coupons were 7-fold lower than for carbon steel beads. Longer incubation times can cause the calculated weight loss corrosion rates to be lower, when most of the weight loss corrosion occurs in the first few days of incubation. The relationship between bronopol concentration (mM) and the electrochemically determined corrosion rate (mm/yr) was also linear with F = 56.75, p = 0.002 and r^2^ = 0.934.

**Table 4:** Summary of results of potentiodynamic polarization scanning for CT coupon WEs incubated at different bronopol concentrations for 24 h. The tabulated data were derived from single incubations.

Bronopol (mM)	E_corr_ (V)	b_c_ (mV/dec)	b_a_ (mV/dec)	i_corr_/A (μA/cm^2^)^a^	Corrosion rate (mm/yr)^b^
0.0	-0.68	-190.9	131.4	1.43	0.016
0.5	-0.59	-517.0	174.8	8.7	0.100
1.0	-0.58	-475	84.13	10	0.115
1.5	-0.58	-280.0	72.3	11	0.126
2.0	-0.57	-296	117	17	0.196
2.5	-0.52	-238	113	23	0.264

^a^Calculated using Eq 3, The reference electrode used was Ag/AgCl/3.5 M KCl

^b^Corrosion rate = 0.0115 mm/yr for (i_corr_/A) = 1 μA/cm^2^

Bronopol is an electrophilic biocide, which reacts with electron rich thiol groups in glutathione or in cysteines in membrane proteins of bacterial cell walls. This can produce reactive oxygen species, which aggressively attack metal surfaces in the absence of other reactive targets like biomass. Hydrolysis of bronopol produces reactive products like formaldehyde and nitrosamines. It photodegrades to tris (2-hydroxymethyl-2-nitropropane-1,3-diol) [7]. High corrosion rates in samples with excess bronopol can be caused by the release of formaldehyde, which is corrosive to carbon steel [23]. The efficacy of biocides is influenced by pH, TDS, salinity, mode of biocidal action and by the presence of other chemicals (stabilizers, reservoir oil, surfactants, antifoams and corrosion inhibitors) used in field operations [24, 25]. Their efficacy is also influenced by temperature [26]. Hence, compatibility tests with the field environment are needed to prevent poor biocide performance [27].

### Profilometric analysis of coiled tubing coupons

**Fig 7:**
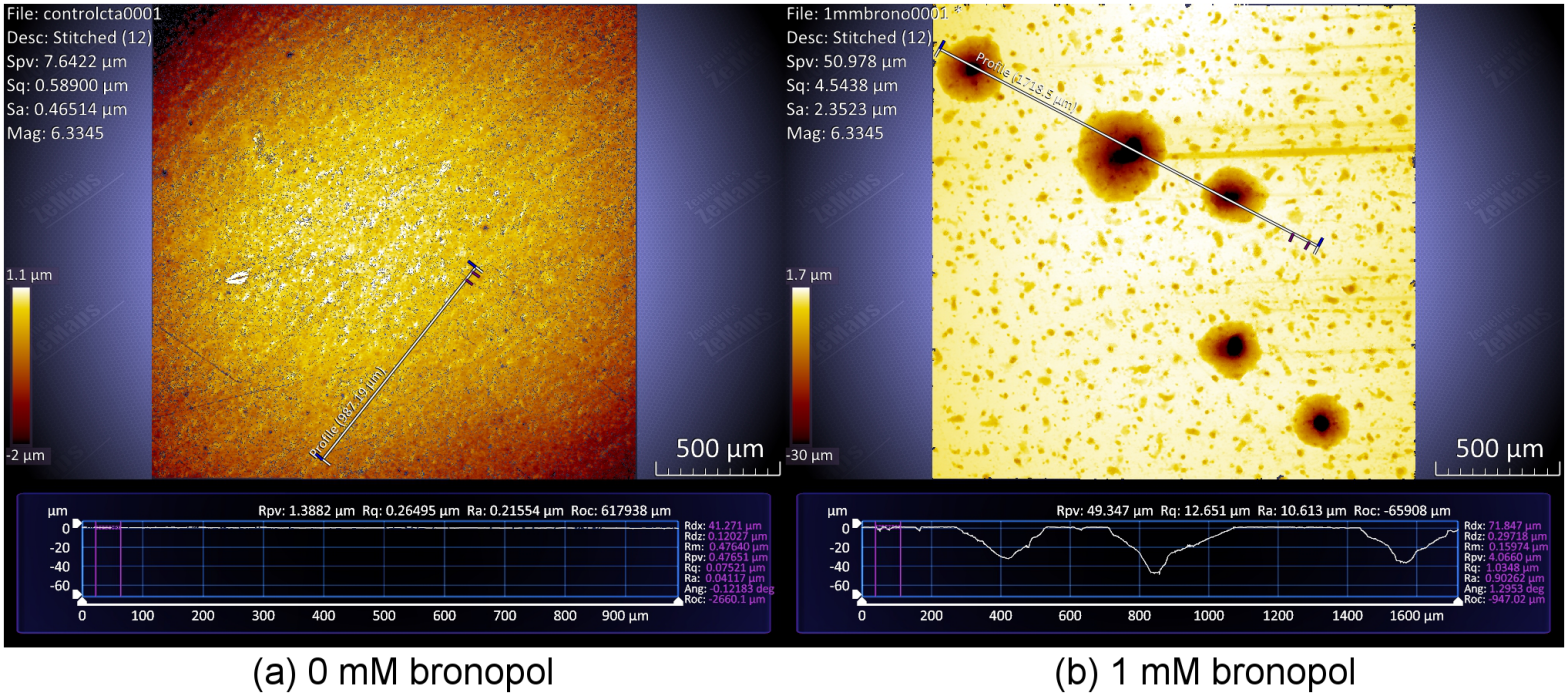
Optical profilometer image and pit depth profile for CT coupon in the (a) absence and (b) presence of bronopol (1 mM) after one week of incubation with N_2_-CO_2_ headspace.

After 1 week of incubation the CT coupons were analyzed using optical profilometer. In the absence of bronopol, the surface of the coupon remained smooth and no pits were observed (Fig 7a). However, the surface of coiled tubing coupons incubated with bronopol (1 mM) showed severe pitting with an average pit depth of 60 μm (Fig 7b). Coiled tubing coupon samples incubated with other bronopol concentrations (0.5, 1.5, 2, 2.5 mM) also showed similar pitting profile (data not shown).

### Microbial community analyses

**Fig 8:**
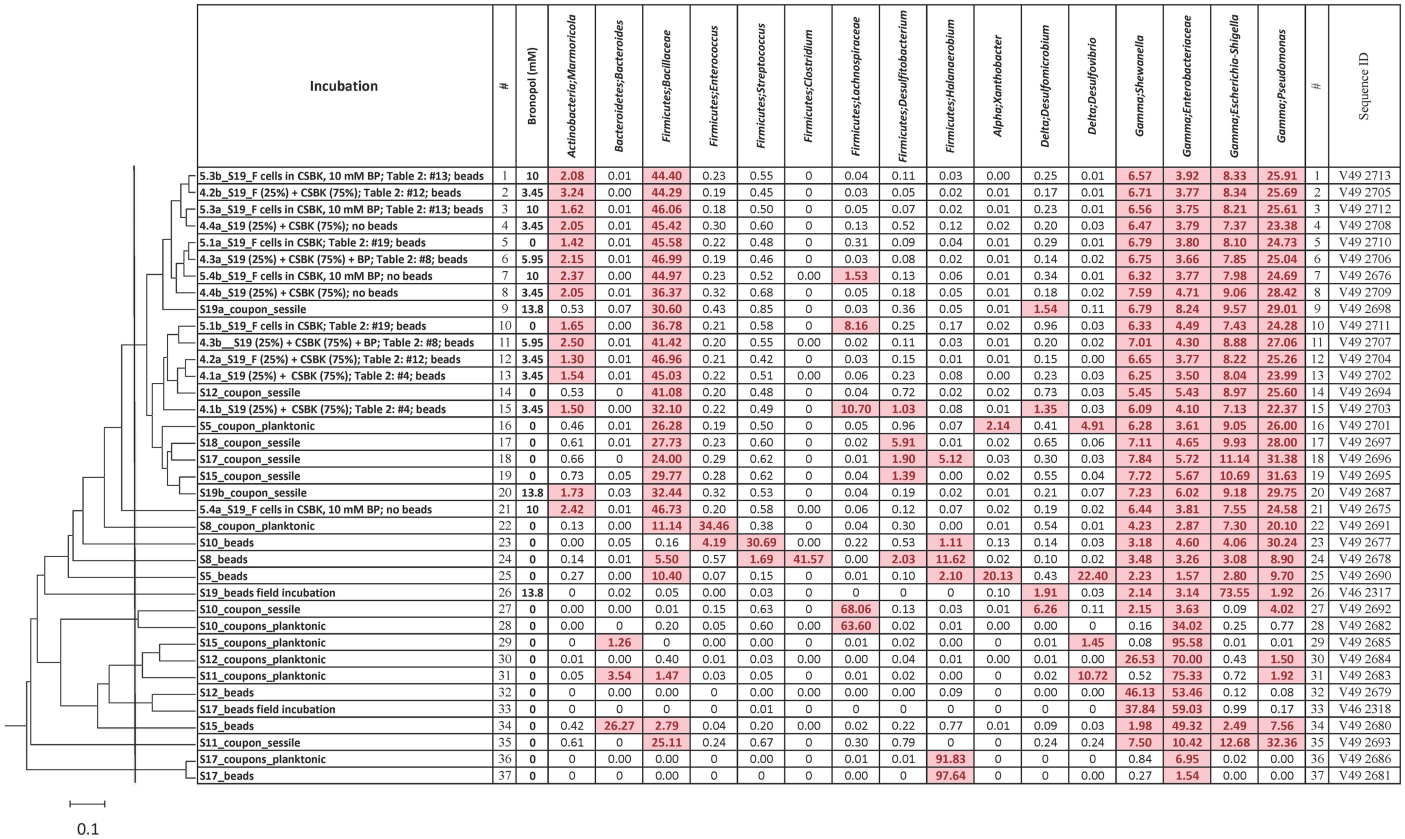
Microbial community compositions derived from Illumina-sequenced amplicons obtained from incubations of field water samples S5 to S19 with carbon steel beads or coiled tubing coupons (entries #9, 14, 16–20 and 22–37) or from incubations with sample S19 (entries #1–8, 10–13, 15, 21). The latter incubations were mostly as described in Table 2. The estimated concentrations of bronopol in these incubations are given. Fractions of predominant taxa (% of total sequence reads) are shown. Fractions in excess of 1% are shaded. A rank identifier (#) and sequence ID are also given. The similarity of community compositions is indicated in a Bray-Curtis dendrogram with the vertical line drawn at 18% sequence divergence (bar is 10% sequence divergence).

The microbial community compositions of field water samples S5 to S20 were determined previously to be dominated by *Gammaproteobacteria* of the genera *Pseudomonas*, *Shewanella*, and *Raoultella* [8]. In the present study, microbial communities in incubations of these field water samples with either carbon steel beads or coiled tubing coupons were also analyzed. In the case of incubations with carbon steel beads, only the planktonic population was analyzed. For incubations with coiled tubing coupons, one of the coupons was scraped to also analyze the sessile population. In addition microbial communities were determined for some of the incubations described in Table 2. Altogether 37 16S rRNA amplicons were obtained and subjected to Illumina sequencing. The microbial community compositions derived for these 37 amplicon libraries are compared in Fig 8.

Incubations of field water samples, which did not contain active bronopol (all except S19), had very diverse microbial communities (Fig 8: entries #9, 14, 16–20 and 22–37). Many of these were dominated by the same *Gammaproteobacteria* identified earlier, i.e. the genera *Pseudomonas* and *Shewanella*, as well as by the genera *Escherichia/Shigella* and the family *Enterobacteriaceae*. However, some of these incubations developed very distinct populations. The planktonic and sessile populations in the incubation of S10 with coupons were dominated by *Firmicutes/Lachnospiraceae* (Fig 8: entries #27 and 28), whereas an incubation of the same field water sample with carbon steel beads (Fig 8: entry #23) was dominated by *Firmicutes/Streptococcus* and *Gammaproteobacteria/Pseudomonas*. The planktonic populations of S17 incubations with beads and coupons were very similar with high fractions of *Firmicutes/Halanaerobium* (Fig 8: entries #36 and 37), which were distinct from the sessile population in the incubation with coupons (Fig 8: entry #18) and from the planktonic population of an incubation, which was started in the field (Fig 8: entry #33). Some incubations with field water samples showed high fractions of *Deltaproteobacteria/Desulfovibrio* or *Deltaproteobacteria/Desulfomicrobium* (e.g. Fig 8: entries #25, 26 and 31). *Desulfomicrobium* is generally associated with oil field reservoir souring by reduction of sulfate to sulfide using different electron donors, including H_2_ [28–30]. *Desulfovibrio* is a commonly found corrosive SRB, which has been used in many laboratory-based studies of microbially-influenced corrosion [31–34].

In contrast to the high diversity of microbial communities in field samples other than S19 incubated with beads or coupons, those in incubations with injection water sample S19 were much less diverse. At 18% sequence divergence fifteen of these were in a single clade; only two (Fig 8: entries #21 and 26) were outside this clade. The S19 clade also contained sessile communities from five other field water samples (Fig 8: entries #14, 16–19). Most incubations of S19 samples contained a high concentration of bronopol (Fig 8). However, in some cases bronopol was removed by filtration and transferring the filter to CSBK medium. However, the communities found in these incubations (Fig 8: entries #5 and 10) did not change. The most reasonable explanation for these results is that there was no growth in any of these incubations because microbes had been killed by the high bronopol concentrations present in the S19 field water sample. The most prominent taxa in the S19 clade were *Firmicutes/Bacillaceae* and *Gammaproteobacteria/Pseudomonas* (Fig 8).

Of the taxa found in Fig 8 *Bacillaceae* and *Pseudomonas* are acid-producing bacteria, which can ferment guar gum polymers used as proppant in fracking operations under the generally anaerobic conditions of shale gas environments. These can also participate in localized corrosion attacks by causing electrochemical alterations of the metal surface [35, 36]. Exopolysaccharide (EPS) formed by *Pseudomonas fluorescens* on steel coupons has been previously studied by Beech et al (1991) and increased corrosion was reported due to formation of biofilms, promoting accumulation of organic acids on the metal surface [37]. Taxa of the phylum *Firmicutes* can also serve as acid-producing bacteria fermenting carbohydrates [38]. In addition to the *Bacillaceae*, this included the genera or families *Enterococcus*, *Streptococcus*, *Clostridium*, *Lachnospiraceae*, *Desulfitobacterium* and *Halanaerobium* (Fig 8). We do not have sufficient understanding of the physiology and corrosion-related properties of these genera to state whether their presence is associated with increased corrosion.

In summary, it appears that microbial communities of coiled tubing field water samples [8] and of coiled tubing field water sample incubations with carbon steel (this study) are dominated by acid-producing bacteria belonging to the *Gammaproteobacteria* and *Firmicutes*. Some samples had very specific community compositions, but the relation of these to corrosion is not clear. Incubations of injection water sample S19 all had very similar community compositions, likely due to lack of growth caused by the high concentration of the biocide bronopol in this sample.

## Conclusions

The high weight loss corrosion rate of carbon steel beads determined for S19 (1.25±0.07 mm/yr), an injection water sample from a coiled tubing operation in a shale gas field, was correlated to the high concentration of active bronopol in this sample (Fig 4). Corrosion rate measurements by weight loss and by electrochemical tests indicated that increasing concentrations of bronopol increased the corrosion rate. Increased pitting was also evident. Hence, overdosing of bronopol should be avoided.

Injection water sample S19 had no viable acid-producing or sulfate-reducing bacteria, which was not surprising in view of the high concentration of biocide detected. Microbial community compositions in incubations for determination of corrosion rates varied little indicating lack of microbial growth caused by the presence of high bronopol concentrations.

The remaining active bronopol concentration in recirculating water in a shale gas operation can be rapidly determined by HPLC, whereas previous dosing of bronopol may be similarly determined as an increase in the bromide concentration. These chemical assays may be better suited to decide whether further addition is needed than assays based on numbers or activity of surviving bacteria. Accurate estimation of the need for more biocide addition in shale gas operations is clearly important, because this protects the environment and prevents unwanted biocide-mediated corrosion.
